# Serum Metabolomic Signatures of Iron Deficiency Anemia: An Untargeted LC‐HRMS‐Based Case–Control Study

**DOI:** 10.1155/anem/6256940

**Published:** 2026-10-05

**Authors:** Metin Demirel, Fatmanur Köktaşoğlu, Halime Dulun Ağaç, Ayşe Zehra Gül, Ufuk Sarıkaya, Ahmet Ceyhan Goren, Cumali Karatoprak, Şahabettin Selek

**Affiliations:** ^1^ Department of Medical Biochemistry Faculty of Medicine Bezmialem Vakif University Istanbul Türkiye bezmialem.edu.tr; ^2^ Department of Biochemistry Sancaktepe Şehit Prof.Dr. İlhan Varank Training and Research Hospital Istanbul Türkiye; ^3^ Vocational School of Health Services Department of Medical Services and Techniques Bezmialem Vakif University Istanbul Türkiye bezmialem.edu.tr; ^4^ Department of Chemistry Gebze Technical University Kocaeli Türkiye gyte.edu.tr; ^5^ Department of Internal Medicine Faculty of Medicine Bezmialem Vakif University Istanbul Türkiye bezmialem.edu.tr

**Keywords:** amino acid metabolism, branched-chain amino acids, iron deficiency anemia, nitric oxide pathway, untargeted metabolomics

## Abstract

**Objective:**

Iron deficiency anemia is primarily evaluated using hematological and iron‐related parameters; however, these markers may not fully reflect the systemic metabolic consequences of impaired oxygen delivery and altered iron availability. This study aimed to characterize serum metabolomic alterations associated with iron deficiency anemia using an untargeted LC‐HRMS approach.

**Materials and Methods:**

Serum samples from 47 individuals with iron deficiency anemia and 50 healthy controls were analyzed by LC‐HRMS in positive and negative ionization modes. Data processing and metabolite annotation were performed using MassCube and MZmine workflows. Multivariate and univariate analyses, covariate‐adjusted models, metabolite set enrichment analysis, correlations with hematologic/iron indices, and internally cross‐validated exploratory ROC analyses were performed.

**Results:**

PCA showed partial separation between iron deficiency anemia and control groups, while OPLS‐DA demonstrated clearer group discrimination. Differential metabolites indicated prominent alterations in amino acid, central carbon, nitrogen‐related, and redox‐associated pathways. The principal amino‐acid/nitrogen‐related differences remained significant after adjustment for age, sex, albumin, and total protein. In the full cohort, several discriminatory metabolites correlated with hemoglobin, ferritin, and transferrin saturation, although these correlations did not remain FDR‐significant within the IDA group alone. Repeated stratified 5‐fold cross‐validation yielded a mean AUC of 0.964 for the three‐metabolite model comprising isoleucine, isonicotinic acid, and phenylalanine.

**Conclusion:**

Untargeted serum metabolomics revealed a distinct metabolic signature in iron deficiency anemia, mainly involving amino acid remodeling, central carbon metabolism, nitrogen balance, and redox‐associated pathways. These findings provide a basis for future targeted and externally validated metabolomics studies.

## 1. Introduction

Iron deficiency anemia (IDA) is characterized by impaired oxygen‐carrying capacity and is routinely evaluated using hematological and iron‐related parameters, including hemoglobin, ferritin, serum iron, TSAT, and total iron‐binding capacity (TIBC) [1, 2]. Although these indices are essential for diagnosis and clinical follow‐up, they do not fully capture the systemic biochemical consequences of IDA. Reduced oxygen delivery, altered iron availability and impaired iron‐dependent enzymatic activity may affect mitochondrial oxidative phosphorylation, redox homeostasis, and substrate utilization, thereby promoting hypoxia‐like metabolic adaptation [3–5]. In this context, metabolomics provides a complementary strategy for evaluating low‐molecular‐weight metabolites that reflect changes in energy metabolism, amino acid turnover, oxidative stress and inflammation‐related metabolic regulation.

Previous metabolomics studies in anemia and IDA have reported alterations in the tricarboxylic acid cycle (TCA‐cycle) intermediates, branched‐chain and aromatic amino acids and metabolites related to mitochondrial and host–microbial metabolism [6–9]. In particular, metabolites such as succinate, isoleucine and phenylalanine may provide mechanistic clues regarding impaired oxidative metabolism, altered fuel utilization and inflammation‐ or hypoxia‐associated metabolic remodeling [10, 11]. However, adult human serum metabolomics data in IDA remain limited and liquid chromatography–high resolution mass spectrometry (LC‐HRMS)–based untargeted studies are still scarce. Therefore, the present study aimed to characterize serum metabolomic alterations in patients with IDA compared with healthy controls using an untargeted LC‐HRMS approach, with particular emphasis on identifying affected metabolic pathways and candidate metabolites that may reflect IDA‐related systemic metabolic adaptation. This study is intended as an exploratory contribution to the metabolomic characterization of IDA rather than a definitive biomarker validation study.

## 2. Materials and Methods

### 2.1. Study Design

This prospective, single‐center case–control study was conducted at Bezmialem Vakif University Faculty of Medicine Hospital using residual serum specimens generated during routine clinical testing that would otherwise have been discarded as biological waste. Participants were enrolled and samples were collected between June 7 and July 9, 2021. Samples with the most complete clinical and laboratory data and adequate residual serum availability were selected for the metabolomics experiment, resulting in a final LC‐HRMS dataset of 47 individuals with IDA and 50 healthy controls. Patients were diagnosed with IDA according to low serum ferritin (Fer < 30 ng/L), low hemoglobin (Hb < 7.7 mmol/L in men and < 7.4 mmol/L in women), low serum iron (Fe < 7.1 μg/L), and high TIBC (> 13.1 μmol/L). Unsaturated iron‐binding capacity (UIBC) was also recorded, and TSAT was calculated as serum iron/TIBC × 100 for descriptive and correlation analyses; TSAT was not used as an additional diagnostic cutoff. Controls were independently selected from eligible healthy participants and were not individually matched to cases by age or sex; group comparability was evaluated statistically.

Individuals with pregnancy, smoking, hypertension, diabetes mellitus, malignancy, chronic inflammatory disease, any other chronic systemic disorder, or malnutrition were excluded to minimize potential metabolic confounding. Malnutrition was operationally defined as BMI < 18.5 kg/m^2^. A validated nutritional screening score and detailed dietary intake questionnaire were not administered. Blood samples were collected after an 8‐h fast and before iron treatment or supplementation. The exact time of day of blood collection was not systematically recorded in the archived study log. Serum samples were aliquoted and stored at −80°C until LC‐HRMS‐based untargeted metabolomic analysis.

The study was conducted in accordance with the Declaration of Helsinki and was approved by the Clinical Research Ethics Committee of Bezmialem Vakif University (No: 2021/286). Written informed consent was obtained from all participants before sample collection.

### 2.2. Sample Preparation

Serum samples were thawed on ice and vortexed gently before extraction. For each sample, 300 μL of serum was mixed with 1.5 mL of cold methanol for protein precipitation and metabolite extraction. The mixture was vortexed thoroughly and incubated on ice. Samples were then centrifuged at 10.000 × g for 30 min at 4°C.

After centrifugation, the clear supernatant was transferred into clean tubes and filtered through 0.22‐μm filters. The filtered extracts were evaporated to dryness using a vacuum concentrator at 60°C under alcohol‐compatible evaporation mode. The dried residues were reconstituted in 100 μL of HPLC‐grade methanol, vortexed, and transferred to LC‐MS vials for analysis.

A pooled quality control sample was prepared by combining 5 μL aliquots from each study sample. The pooled QC sample was injected after every 20 study samples. Methanol blanks were also included in the analytical sequence.

### 2.3. LC‐HRMS Analysis

Untargeted metabolomic profiling was performed using a Thermo Fisher Scientific Q Exactive Plus Hybrid Quadrupole‐Orbitrap mass spectrometer equipped with a heated electrospray ionization source and coupled to a liquid chromatography system. Chromatographic separation was achieved using a Troyasil C18TNE column, 5 μm, 50 × 2.1 mm, maintained at 40°C.

The mobile phase consisted of solvent A, water containing 0.1% formic acid, and solvent B, methanol containing 0.1% formic acid. The injection volume was 5 μL and the flow rate was 0.3 mL/min. The total chromatographic runtime was 18 min. A gradient elution program was applied as follows: 0–1 min, 5% B; 1–14 min, linear increase to 95% B; 14–16 min, 95% B; 16.1 min, return to 5% B; and 16.1–18 min, re‐equilibration at 5% B.

Mass spectrometric data were acquired in both positive and negative ionization modes. Full‐scan MS data were collected over an m/z range of 50–900. The HESI source parameters were as follows: sheath gas flow rate, 45 arbitrary units; auxiliary gas flow rate, 10 arbitrary units; spray voltage, approximately 3.8 kV; capillary temperature, 320°C; auxiliary gas heater temperature, 320°C; and S‐lens RF level, 50. Data‐dependent MS/MS acquisition was performed for metabolite annotation. Study samples, pooled QC samples and methanol blanks were analyzed within the same analytical workflow.

Raw LC‐HRMS data were processed using the MassCube Python package, Version 1.2.5, including raw data import, mass trace detection, feature detection, chromatographic peak construction, alignment, gap filling and final feature table generation [12]. Key preprocessing parameters included MS1 and MS2 absolute intensity thresholds of 30,000 and 10,000, respectively; mass tolerances of 0.01 Da for MS1, 0.015 Da for MS2 and 0.01 Da for alignment; a retention time alignment tolerance of 0.3 min; and an MS/MS spectral similarity threshold of 0.7.

Raw LC‐MS/MS data were also processed using MZmine Version 4.9 to support feature detection, feature alignment, feature list generation and metabolite annotation [13].

Metabolite annotation was performed using accurate mass, retention time behavior, isotope pattern, adduct information, and MS/MS fragmentation spectra when available. Experimental MS/MS spectra were compared with public metabolomics databases and spectral libraries, including HMDB, MoNA, and related compound databases. Annotation confidence was assigned according to the Metabolomics Standards Initiative framework. Since authentic reference standards were not used for all annotated metabolites, the reported metabolites were considered putatively annotated, unless otherwise confirmed.

### 2.4. Statistical Analysis

The final annotated metabolomics matrix contained 94 metabolites across 97 samples and was analyzed using MetaboAnalyst 6.0 and complementary statistical analyses [14]. Each sample was normalized to its median signal intensity, followed by square‐root transformation and autoscaling. The final matrix contained no blank or NA entries; zero‐intensity values were retained and no additional imputation was performed. As a sensitivity analysis for feature presence, metabolites were additionally required to be detected in at least 80% of samples in at least one study group.

Principal component analysis (PCA) was performed to evaluate the overall distribution of serum metabolomic profiles and visualize sample clustering. The global difference in metabolomic profiles between the IDA and Control groups was assessed using PERMANOVA with 999 permutations.

Orthogonal partial least squares‐discriminant analysis (OPLS‐DA) was performed to evaluate supervised separation between the IDA and control groups. Model performance was assessed using R^2^X, R^2^Y, and Q^2^ values, and permutation testing was used to evaluate model robustness.

Univariate comparisons were performed using two‐sample *t* tests on the transformed data. Fold change (FC) was calculated to evaluate the magnitude and direction of group differences. Benjamini–Hochberg (BH) false discovery rate correction was applied uniformly across all 94 metabolites. Differential metabolites were defined as those with *q* < 0.05 and |log_2_FC| ≥ 0.5. To assess potential confounding, covariate‐adjusted linear models were fitted separately for each autoscaled metabolite. The primary adjusted model included group, age, and sex. A nutritional‐proxy sensitivity model additionally included serum albumin and total protein. Group‐level BMI data were available and did not differ between groups; however, participant‐level BMI values were not available in the metabolomics‐linked dataset used for the covariate reanalysis and therefore could not be entered into these models. BH correction was applied across the 94 group coefficients within each adjusted model.

Differential metabolites were visualized using volcano plots. Relationships between metabolite abundance and hematologic/iron indices (hemoglobin, hematocrit, MCV, RDW, serum iron, ferritin, UIBC, TIBC, and TSAT) were assessed using Spearman rank correlation. BH correction was applied separately across the 94 metabolites for each clinical variable. Correlations were evaluated in the full cohort and, as a sensitivity analysis, within the IDA group alone. Receiver operating characteristic (ROC) analysis was used to evaluate exploratory discriminatory performance. In addition to apparent AUCs, internal validation was performed using 100 repeats of stratified 5‐fold cross‐validation; predictor standardization and logistic model fitting were performed within training folds. A nested sensitivity analysis additionally repeated feature selection within each training fold before fitting a three‐feature model.

Pathway enrichment analysis was performed using the annotated metabolite list to identify metabolic pathways potentially affected in IDA. Pathway interpretation was based on KEGG‐associated metabolic pathways and metabolite set enrichment results. This study was exploratory and used the available eligible cohort. With 47 IDA participants and 50 controls, a two‐sided two‐sample comparison at *α* = 0.05 provided approximately 80% power to detect a standardized between‐group difference of Cohen’s *d* ˜ 0.58. The study was not powered to detect small metabolite effects after extensive multiple‐testing correction; therefore, biomarker and ROC analyses were considered exploratory and require external validation.

## 3. Results

LC‐MS‐based untargeted metabolomic analysis was performed on serum samples from the IDA and Control groups. After data processing and metabolite annotation, 94 annotated metabolites or metabolite‐related features were included in the final data matrix, comprising 47 IDA and 50 Control samples. Demographic, hematologic, and biochemical characteristics are summarized in Table 1. Age and sex distribution did not differ significantly between groups (*p* = 1.000 and *p* = 0.195, respectively). BMI (24.4 ± 3.4 vs 24.5 ± 5.0 kg/m^2^) and fasting glucose (89.7 ± 8.6 vs 88.9 ± 9.0 mg/dL) were also comparable between IDA and Control groups, respectively. Clinical creatinine, urea, total protein, and albumin concentrations were comparable (all *p* > 0.05). In contrast, hemoglobin, hematocrit, MCV, MCH, MCHC, serum iron, ferritin, and TSAT were markedly lower in IDA, whereas RDW, UIBC, TIBC, and platelet counts were higher (all *p* < 0.001).

**TABLE 1:** Demographic, hematologic, and biochemical characteristics of the study groups.

Variable	IDA	Control	*p*
Age (years)	40.00 [28.50–48.00]	40.00 [31.00–56.50]	1.000
Female sex, *n* (%)	41 (87.2%)	38 (76.0%)	0.195
Hemoglobin (g/dL)	9.16 [7.77–10.72]	13.29 [12.61–14.11]	< 0.001
Hematocrit (%)	30.50 [25.68–34.21]	39.97 [38.18–42.32]	< 0.001
MCV (fL)	72.37 [64.38–77.83]	87.39 [85.00–91.53]	< 0.001
MCH (pg)	21.97 [18.55–24.98]	29.44 [28.40–30.63]	< 0.001
MCHC (g/dL)	30.26 [29.31–31.88]	33.26 [32.45–34.02]	< 0.001
RDW (%)	15.37 [14.23–16.04]	12.59 [12.21–13.12]	< 0.001
Serum iron	17.00 [14.00–28.00]	86.00 [71.50–111.50]	< 0.001
Ferritin	2.99 [1.79–7.88]	38.11 [18.45–66.84]	< 0.001
UIBC	377.00 [333.00–405.00]	215.00 [183.50–258.50]	< 0.001
TIBC	391.00 [361.00–434.00]	317.00 [261.50–349.00]	< 0.001
Transferrin saturation (%)	4.50 [3.66–7.82]	28.34 [25.38–35.94]	< 0.001
Platelets (× 10^3^/μL)	277.00 [228.00–337.50]	237.00 [196.25–267.25]	< 0.001
Creatinine	0.77 [0.71–0.88]	0.81 [0.75–0.89]	0.192
Urea	23.00 [18.00–30.50]	24.00 [19.00–28.00]	0.905
Total protein	8.10 [7.45–8.50]	7.80 [7.40–8.00]	0.062
Albumin	4.60 [4.15–4.90]	4.65 [4.40–4.90]	0.263

*Note:* Continuous variables are presented as median [interquartile range] and were compared using the Mann–Whitney *U* test. Sex distribution was compared using Fisher’s exact test. TSAT was calculated as serum iron/TIBC × 100.

Routine clinical creatinine concentrations did not differ significantly between groups (median 0.77 [0.71–0.88] in IDA vs 0.81 [0.75–0.89] in controls; *p* = 0.192), whereas the relative abundance of the creatinine feature detected by untargeted LC‐HRMS was higher in IDA (FC = 1.558, BH *q* = 1.35 × 10^−6^). These measurements were generated by different analytical approaches and should not be interpreted as directly equivalent quantitative measurements.

PCA was performed to evaluate the global distribution of serum metabolomic profiles between the IDA and Control groups (Figure 1). In the PCA score plot, PC1 and PC2 explained 23.2% and 15.4% of the total variance, respectively. The IDA and Control groups showed partial separation in the PCA space. PERMANOVA showed a significant difference between groups (*F* = 22.838, *R* ^2^ = 0.19381, *p* = 0.001; 999 permutations). OPLS‐DA was then applied to evaluate group separation using a supervised multivariate model. The OPLS‐DA score plot showed separation between the IDA and Control groups along the predictive component (Figure 1). The predictive component explained 12.0% of the variance, while the orthogonal component explained 16.3%. Permutation testing showed empirical *p* values of *p* < 0.001 for both Q^2^ and R^2^Y based on 1000 permutations and *p* < 5 × 10^−4^ for both Q^2^ and R^2^Y based on 2000 permutations.

**FIGURE 1:**
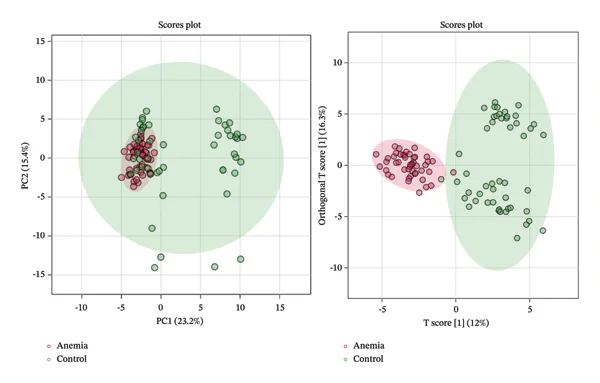
PCA and OPLS‐DA score plots of LC‐MS‐based serum metabolomic profiles.

Volcano plot analysis identified 36 metabolites with differential abundance between the IDA and Control groups based on FC and BH‐adjusted *q* values (Figure 2). The most significantly altered metabolites were isoleucine (FC = 2.3189, log_2_FC = 1.2134, *q* = 4.41 × 10^−13^), piperidine (FC = 2.3073, log_2_FC = 1.2062, *q* = 9.85 × 10^−13^), isonicotinic acid (FC = 0.3955, log_2_FC = −1.3383, *q* = 4.07 × 10^−11^), galactaric acid (FC = 0.5115, log_2_FC = −0.9671, *q* = 3.15 × 10^−9^), and phenylalanine (FC = 1.8433, log_2_FC = 0.8823, *q* = 5.70 × 10^−9^). Metabolites with higher abundance in the IDA group included isoleucine, piperidine, phenylalanine, ketoleucine, N6,N6,N6‐trimethyl‐L‐lysine, creatinine, asymmetric dimethylarginine (ADMA), methylhistidine, uric acid, theobromine, L‐arginine, pyroglutamic acid, gentianine, trigonelline, oxoglutaric acid, and diethyl phthalic acid. Metabolites with lower abundance included isonicotinic acid, galactaric acid, 4‐guanidinobutanoic acid, p‐anisic acid, imidazoleacetic acid, oleamide, isofraxidin, azelaic acid, 2‐hydroxyphenethylamine, L‐homoserine, 2,4‐dinitrophenol, chrysophanol, glutamic acid, morpholine, 1‐methylnicotinamide, 4‐hydroxy‐2‐butenoic acid gamma‐lactone, jasmonic acid, 4‐pyridoxic acid, kynurenic acid, and bisphenol S. In the final 94 × 97 matrix, 545 zero‐intensity cells (5.98%) were present and no blank/NA cells remained. Eighty‐seven of 94 metabolites were detected in at least 80% of samples in at least one group; applying this presence criterion as a sensitivity filter retained 34 of the 36 differential metabolites and did not alter the principal amino‐acid/nitrogen‐related findings.

**FIGURE 2:**
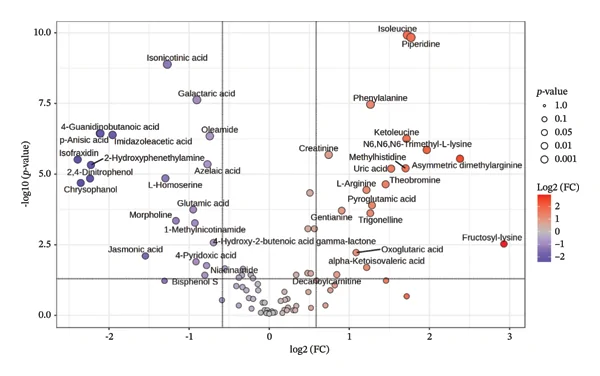
Volcano plot of differential serum metabolites between the IDA and Control groups. The *x*‐axis represents log_2_ FC and the *y*‐axis represents −log_10_ *p* value. Metabolites on the right side of the plot show higher abundance in the IDA group, while metabolites on the left side show lower abundance in the IDA group. Labeled metabolites indicate selected features with prominent group differences.

Covariate adjustment did not materially attenuate the principal amino‐acid/nitrogen signature (Table 2). Isoleucine, phenylalanine, methylhistidine, ketoleucine, creatinine, uric acid, ADMA, L‐arginine, isonicotinic acid, and piperidine remained FDR‐significant after adjustment for age, sex, albumin, and total protein. Oxoglutaric acid remained significant in the age‐ and sex‐adjusted model (*q* = 0.015) but did not remain FDR‐significant after additional adjustment for the nutritional proxies (*q* = 0.076).

**TABLE 2:** Covariate‐adjusted group effects for reviewer‐highlighted discriminatory metabolites.

Metabolite	FC	Unadjusted *q*	*β*: age + sex	*q*: age + sex	*β*: age + sex + albumin + TP	*q*: Nutrition model
Isoleucine	2.319	4.41 × 10^−13^	1.347	1.99 × 10^−12^	1.366	2.14 × 10^−10^
Phenylalanine	1.843	5.70 × 10^−9^	1.137	1.93 × 10^−8^	1.115	1.01 × 10^−6^
Methylhistidine	1.998	5.49 × 10^−6^	0.941	1.03 × 10^−5^	0.963	7.32 × 10^−5^
Ketoleucine	1.877	2.64 × 10^−7^	1.015	9.14 × 10^−7^	1.056	7.98 × 10^−6^
Creatinine	1.558	1.35 × 10^−6^	0.990	2.75 × 10^−6^	0.899	1.58 × 10^−4^
Uric acid	1.661	5.49 × 10^−6^	0.923	1.22 × 10^−5^	0.930	1.28 × 10^−4^
Oxoglutaric acid	3.697	0.008	0.557	0.015	0.457	0.076
Asymmetric dimethylarginine	1.776	2.01 × 10^−6^	0.984	2.85 × 10^−6^	1.036	1.68 × 10^−5^
L‐Arginine	1.518	3.99 × 10^−5^	0.842	8.15 × 10^−5^	0.842	5.95 × 10^−4^
Isonicotinic acid	0.395	4.07 × 10^−11^	−1.298	5.17 × 10^−11^	−1.249	1.93 × 10^−8^
Piperidine	2.307	9.85 × 10^−13^	1.321	4.46 × 10^−12^	1.345	3.37 × 10^−10^

*Note:* β denotes the standardized group coefficient for IDA versus Control after median normalization, square‐root transformation, and autoscaling. The first adjusted model included group, age, and sex. The nutritional‐proxy model additionally included serum albumin and total protein. BH correction was applied across all 94 metabolite group coefficients within each model.

Neither isoleucine nor phenylalanine showed significant full‐cohort correlations with albumin or total protein (all *p* > 0.14 for isoleucine and *p* > 0.28 for phenylalanine). Methylhistidine was likewise not significantly associated with albumin (*ρ* = 0.063, *p* = 0.539) or total protein (*ρ* = 0.169, *p* = 0.097) in the full cohort. Importantly, the IDA–Control differences in isoleucine (*q* = 2.14 × 10^−10^), phenylalanine (*q* = 1.01 × 10^−6^), and methylhistidine (*q* = 7.32 × 10^−5^) remained FDR‐significant after simultaneous adjustment for age, sex, albumin, and total protein, supporting persistence of the principal amino‐acid signature after accounting for the available nutritional proxies.

Metabolite set enrichment analysis was performed using the differential metabolite profile (Figure 3). The top enriched metabolite sets were histidine metabolism; valine, leucine and isoleucine degradation; phenylalanine and tyrosine metabolism.

**FIGURE 3:**
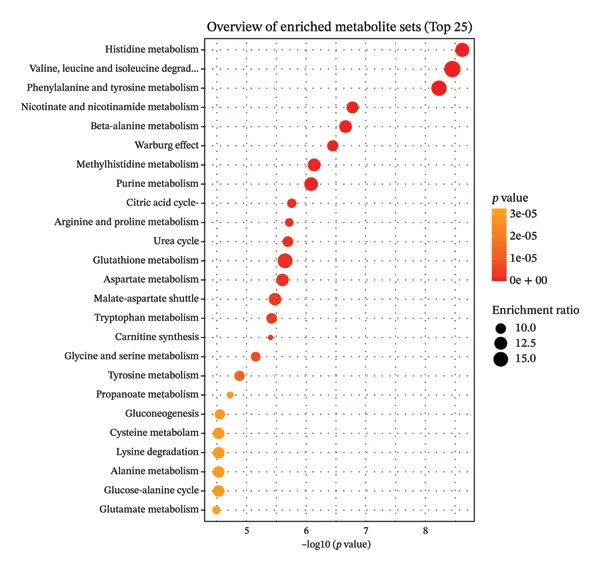
Metabolite set enrichment analysis of differential LC‐MS metabolites. The dot plot shows the top 25 enriched metabolite sets. The *x*‐axis represents −log_10_ *p* value. Dot size represents the enrichment ratio and dot color represents the *p* value.

Relationships between discriminatory metabolites and clinical iron/hematologic indices were subsequently examined. In the full cohort, isoleucine correlated inversely with hemoglobin (*ρ* = −0.644, *q* = 5.49 × 10^−11^), ferritin (*ρ* = −0.611, *q* = 2.89 × 10^−9^), and TSAT (*ρ* = −0.656, *q* = 3.40 × 10^−10^). Phenylalanine showed similar inverse associations with hemoglobin (*ρ* = −0.542, *q* = 1.89 × 10^−7^), ferritin (*ρ* = −0.494, *q* = 5.53 × 10^−6^), and TSAT (*ρ* = −0.584, *q* = 6.88 × 10^−8^), and methylhistidine was likewise inversely associated with major iron‐status indices. In contrast, isonicotinic acid showed positive associations with hemoglobin, ferritin, and TSAT. However, no metabolite–clinical correlation remained FDR‐significant when the analysis was restricted to participants with IDA, indicating that the strongest full‐cohort associations primarily reflected the biochemical contrast between IDA and Control groups rather than a robust within‐IDA severity gradient.

ROC analysis was performed for selected metabolites and the combined model comprising isoleucine, isonicotinic acid, and phenylalanine (Figure 4). The apparent AUCs were 0.938 for isoleucine, 0.938 for piperidine, 0.910 for isonicotinic acid, and 0.820 for phenylalanine; the apparent combined‐model AUC was 0.965. Internal validation using 100 repeats of stratified 5‐fold cross‐validation yielded mean AUCs of 0.933 for isoleucine, 0.928 for piperidine, 0.898 for isonicotinic acid, and 0.814 for phenylalanine. The fixed three‐metabolite model retained a mean cross‐validated AUC of 0.964 (SD = 0.004; 2.5th–97.5th percentile, 0.955–0.970). In a stricter nested sensitivity analysis in which feature ranking was repeated within each training fold, the mean AUC was 0.955 (SD = 0.010; 2.5th–97.5th percentile, 0.933–0.967), indicating that the high discriminatory performance was not solely attributable to resubstitution on the full dataset. These ROC findings remain exploratory because no external validation cohort was available.

**FIGURE 4:**
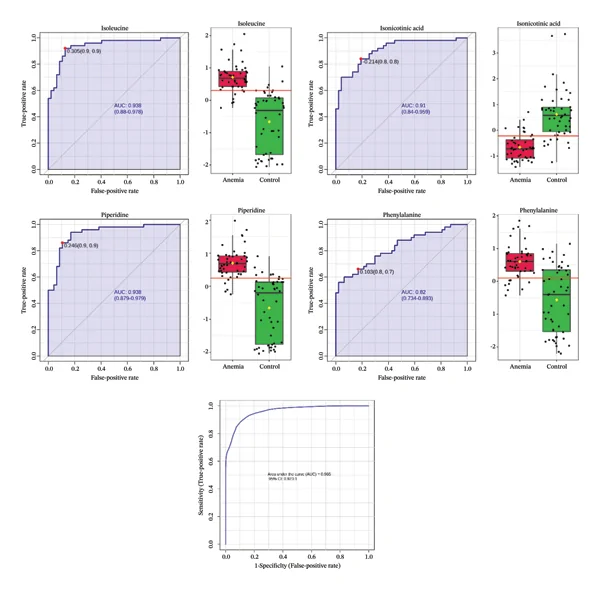
Apparent ROC curves and boxplots for selected metabolites and the combined ROC model. Internal cross‐validation results are reported in the text.

## 4. Discussion

In this LC‐HRMS‐based untargeted serum metabolomics study, 94 annotated metabolites or metabolite‐related features were evaluated in individuals with IDA and healthy controls. The overall metabolic pattern suggests that IDA is accompanied by systemic biochemical remodeling, particularly involving amino acid metabolism, mitochondrial/intermediary metabolism and redox‐related pathways.

A central finding of this study was the alteration of amino acid‐related metabolites, particularly increased isoleucine and phenylalanine. This pattern is biologically plausible because reduced oxygen delivery in IDA may alter mitochondrial oxidative metabolism and increase reliance on alternative energetic substrates. Previous metabolomic studies in IDA and iron deficiency have similarly reported disturbances in amino acid metabolism, including branched‐chain amino acids and aromatic amino acid‐related pathways [7, 8]. In the present dataset, increased isoleucine and ketoleucine may reflect altered branched‐chain amino acid catabolism, whereas increased phenylalanine may indicate impaired aromatic amino acid utilization under metabolic stress. Although these changes cannot be attributed to a single pathway in a cross‐sectional untargeted design, their concordance with IDA‐related and hypoxia‐related metabolomic literature supports a model of altered amino acid turnover and mitochondrial fuel utilization.

Because isoleucine, phenylalanine, and methylhistidine can be influenced by dietary protein intake and nutritional status, these findings require consideration of nutritional confounding. Mean BMI was virtually identical between the IDA and Control groups (24.4 ± 3.4 vs 24.5 ± 5.0 kg/m^2^), and none of these three metabolites showed a significant full‐cohort correlation with albumin or total protein. Their IDA–Control differences also remained FDR‐significant after adjustment for age, sex, albumin, and total protein. These results support robustness to the available nutritional indicators. Nevertheless, detailed dietary intake, recent meat/protein consumption, validated nutritional screening scores, vitamin B12, and folate were not measured, and participant‐level BMI could not be linked to the metabolomics matrix for adjusted modeling; residual nutritional confounding therefore cannot be excluded.

The observed changes in oxoglutaric acid and other energy‐related metabolites suggest perturbation of central carbon metabolism. Iron is required for several mitochondrial enzymes and electron transport chain components; therefore, impaired iron availability and reduced oxygen‐carrying capacity may jointly affect oxidative phosphorylation and TCA‐cycle activity. Prior studies have linked iron deficiency and IDA with altered TCA‐cycle intermediates, mitochondrial dysfunction and a metabolic shift toward less efficient energy production [3, 7, 9]. However, although oxoglutaric acid remained significant after age and sex adjustment, it did not remain FDR‐significant after additional adjustment for albumin and total protein (*q* = 0.076). Its increase should therefore be regarded as a less robust, hypothesis‐generating observation rather than a stand‐alone marker of IDA‐related TCA‐cycle remodeling.

The concurrent alteration of asymmetric dimethylarginine and L‐arginine suggests that the arginine‐nitric oxide pathway may contribute to IDA‐associated metabolic adaptation. ADMA is an endogenous competitive inhibitor of nitric oxide synthase, whereas L‐arginine is the substrate for nitric oxide production, which is essential for endothelial function, vasodilation, microvascular perfusion and vascular adaptation to impaired oxygen delivery [15–17]. Increased ADMA may reduce nitric oxide bioavailability and has been linked to endothelial dysfunction, oxidative stress, inflammatory signaling and impaired hypoxia‐related vascular responses [18–20]. In this context, the increase in both ADMA and L‐arginine should not be interpreted simply as enhanced nitric oxide production. Rather, it may reflect altered substrate availability, impaired downstream utilization, or compensatory responses to reduced nitric oxide signaling, consistent with the concept that the L‐arginine/ADMA balance may be more informative than either metabolite alone.

Alterations in creatinine, methylhistidine and uric acid further support the presence of systemic metabolic stress rather than a single organ‐specific disturbance. Creatinine is closely related to creatine/phosphocreatine turnover and renal clearance, while methylhistidine is commonly considered a marker of myofibrillar protein breakdown and muscle protein turnover. Importantly, routine clinical creatinine concentrations did not differ between groups, whereas the relative untargeted LC‐HRMS creatinine feature was higher in IDA. Because these measurements were generated by different analytical approaches, the LC‐HRMS feature should not be interpreted as directly equivalent to a quantitative clinical creatinine assay. Increased uric acid may reflect altered purine catabolism, ATP degradation, oxidative stress and renal handling, all of which may be relevant under conditions of impaired oxygen delivery and mitochondrial stress [21–23]. These metabolites should therefore be interpreted as components of a broader metabolic pattern involving nitrogen metabolism, muscle‐related protein turnover, purine metabolism and redox balance rather than as direct evidence of renal or muscular dysfunction.

The decrease in isonicotinic acid and other pyridine‐related or diet/microbiota‐associated metabolites may also be relevant. Isonicotinic acid is not a classical endogenous biomarker of IDA and its biological interpretation requires caution because it may reflect exogenous exposure, diet, microbial metabolism, or nicotinate/nicotinamide‐related metabolic processes. However, the broader decrease in several metabolites with potential dietary, microbial, or xenobiotic origins is compatible with emerging evidence that iron deficiency and IDA may influence host–microbiota metabolic interactions [6, 7, 24]. Because detailed dietary intake and microbiome data were not available in the present study, these findings should be regarded as hypothesis‐generating and should be evaluated in future studies integrating metabolomics with nutritional and microbiome profiling.

The correlation analysis further linked the metabolomic pattern to conventional IDA biochemistry. Isoleucine, phenylalanine, methylhistidine, and several other discriminatory metabolites were associated with hemoglobin, ferritin, and TSAT across the full cohort. Nevertheless, no metabolite–clinical correlation remained significant after FDR correction when analysis was restricted to the IDA group. Thus, the full‐cohort correlations should not be interpreted as demonstrating a continuous relationship between metabolite abundance and IDA severity; they appear to be driven largely by the strong biochemical separation between cases and controls.

The ROC analysis identified isoleucine, isonicotinic acid and phenylalanine as metabolites with discriminatory potential between IDA and control groups. Importantly, repeated stratified 5‐fold cross‐validation produced results close to the apparent estimates, with a mean AUC of 0.964 for the fixed three‐metabolite model. A nested feature‐selection sensitivity analysis yielded a mean AUC of 0.955, reducing concern that the observed discrimination was solely due to resubstitution or feature‐selection leakage. Nevertheless, the models were derived and internally validated within a modest single dataset; they should therefore be regarded as exploratory and require evaluation in an independent external cohort before any diagnostic interpretation.

This study has several strengths, including its prospective single‐center sampling framework, use of an untargeted LC‐HRMS approach, integration of multivariate and univariate analyses, covariate‐adjusted sensitivity models, correlation with clinical iron indices, and internal cross‐validation of exploratory ROC models. Nevertheless, several limitations should be considered. The single‐time‐point case–control design does not allow causal inference, and the moderate sample size together with the absence of external validation limits generalizability. Detailed dietary intake, recent meat/protein consumption, socioeconomic information, a validated nutritional screening score, vitamin B12 and folate status, and menstrual status were not collected. Although group‐level BMI was available and did not differ between groups, participant‐level BMI could not be linked to the metabolomics matrix for covariate modeling. C‐reactive protein was not measured; therefore, inflammation‐related effects on ferritin interpretation could not be formally assessed. All samples were collected after an 8‐h fast, but the exact time of day was not systematically recorded. In addition, the underlying etiologies of IDA were not systematically characterized; potential metabolic heterogeneity related to heavy menstrual bleeding, dietary iron deficiency, gastrointestinal blood loss, malabsorption including celiac disease, or other causes could therefore not be assessed. Finally, untargeted LC‐HRMS annotations and relative feature abundances should be confirmed in future targeted quantitative studies using authentic standards.

## 5. Conclusions

This study demonstrates that IDA is associated with a distinct serum metabolomic signature characterized mainly by alterations in amino acid metabolism, central carbon metabolism, nitrogen‐related pathways, and redox‐associated metabolites. The principal differences in isoleucine, phenylalanine, methylhistidine, ADMA, L‐arginine, creatinine, uric acid, and isonicotinic acid persisted after adjustment for age, sex, and available nutritional proxies, while oxoglutaric acid was less robust to nutritional‐proxy adjustment. The findings expand the biochemical perspective of IDA beyond routine hematologic parameters and provide a basis for future targeted metabolomics studies with prospectively standardized nutritional assessment and external validation.

## Author Contributions

Conceptualization: Cumali Karatoprak, Şahabettin Selek; methodology: Metin Demirel, Ahmet Ceyhan Goren, Şahabettin Selek; formal analysis: Metin Demirel, Ufuk Sarikaya; investigation: Fatmanur Köktaşoğlu, Halime Dulun Ağaç, Ayşe Zehra Gül; resources: Cumali Karatoprak, Ahmet Ceyhan Goren, Şahabettin Selek; data curation: Metin Demirel, Fatmanur Köktaşoğlu, Halime Dulun Ağaç; writing–original draft: Metin Demirel; writing–review and editing: Metin Demirel, Fatmanur Köktaşoğlu, Halime Dulun Ağaç, Ayşe Zehra Gül, Ufuk Sarikaya, Ahmet Ceyhan Goren, Cumali Karatoprak, Şahabettin Selek; visualization: Metin Demirel; Supervision: Cumali Karatoprak, Şahabettin Selek; project administration: Şahabettin Selek; funding acquisition: Şahabettin Selek.

## Funding

The study was conducted with the support and funding provided by the Scientific Research Projects Unit of Bezmialem Vakif University (20210802).

## Disclosure

All authors have read and approved the final version of the manuscript. Metin Demirel had full access to all of the data in this study and takes complete responsibility for the integrity of the data and the accuracy of the data analysis.

## Ethics Statement

The study was conducted in accordance with the Declaration of Helsinki and was approved by the Clinical Research Ethics Committee of Bezmialem Vakif University (No: 2021/286).

## Consent

Written informed consent was obtained from all participants before sample collection.

## Conflicts of Interest

The authors declare no conflicts of interest.

## Data Availability

The data supporting the findings of this study are available from the corresponding author upon reasonable request.
